# Dapagliflozin in acute heart failure management: a systematic review and meta-analysis of safety and effectiveness

**DOI:** 10.1186/s12872-024-04412-x

**Published:** 2024-12-28

**Authors:** Adarsh Raja, Mata-e-Alla Dogar, Sandesh Raja, Muhammad Hamza Shuja, Shafin Bin Amin, Muskan Khelani, Urooj Fatima, Aiman Soomro, Ayesha Habiba, Iqra Mustafa, Rakhshan Zulfiqar, Muhammad Sohaib Asghar

**Affiliations:** 1 Department of Medicine, Shaheed Mohtarma Benazir Bhutto Medical College Lyari, Karachi, Pakistan; 2 Department of Medicine, Dow University of Health Sciences, Karachi, Pakistan; 3 Department of Internal Medicine, AdventHealth Sebring, Sebring, FL USA; 4 Department of Internal Medicine, Division of Nephrology and Hypertension, Mayo Clinic, Rochester, MN USA

**Keywords:** Dapagliflozin, Acute heart failure, In-hospital cardiovascular mortality, SGLT2i, Meta analysis

## Abstract

**Background:**

Acute Heart Failure (AHF) presents as a serious pathophysiological disease with significant morbidity and mortality rates, requiring immediate medical intervention. Traditional treatment involves diuretics and vasodilators, but a subset of patients develop resistance due to acute cardiorenal syndrome. Dapagliflozin, categorized as a sodium-glucose cotransporter-2 inhibitor (SGLT2i), has emerged as a promising therapy for AHF, demonstrating substantial benefits in reducing both mortality and morbidity among patients. The purpose of this meta-analysis and systematic review is to determine dapagliflozin’s safety and efficacy in AHF patients.

**Methods:**

In accordance with PRISMA guidelines, we conducted a systematic search across several databases (PubMed, Science Direct, and Cochrane Library) up to June 2024 to identify randomized controlled trials (RCTs) that compared dapagliflozin with control treatments in patients with AHF. Key outcomes of interest included In-Hospital Cardiovascular mortality rates, duration of hospitalization, and instances of in-hospital worsening. Data extraction and quality assessment adhered to established protocols and the results were evaluated using Review Manager (RevMan Version 5.4.1) The assessment of bias risk follows the principles established in the Cochrane Handbook for Systematic Reviews and Meta-Analysis.

**Results:**

Five RCTs comprising 912 patients met the inclusion criteria. Dapagliflozin significantly reduced In-Hospital Cardiovascular mortality (RR 0.56, 95% CI 0.36–0.88, *p* = 0.01, I²=26%) and 30-day hospital readmissions (RR 0.73, CI 0.54–0.99, *p* = 0.05, I²=7%). However, dapagliflozin did not significantly affect the length of hospital stay (MD -0.11, CI -0.73-0.51, *p* = 0.72, I²=60%) or the incidence of hypotension (RR 0.82, CI 0.36–1.84, *p* = 0.63, I²=0%). A significant weight change was observed (MD 0.93, CI 0.03–1.83, *p* = 0.04, I²=95%), which was resolved upon sensitivity analysis (MD 1.34, CI 1.02–1.66, *p* < 0.0001, I²=0%). No significant effects were found for worsening renal failure or changes in GFR in this study.

**Conclusion:**

Dapagliflozin appears to be beneficial in reducing In-Hospital Cardiovascular mortality and 30-day hospital readmissions in AHF patients. Although it demonstrates potential, additional research is needed to establish its significance in AHF management. Further investigation with larger sample sizes, different doses, and comprehensive safety and cost-effectiveness is imperative to thoroughly evaluate the safety and clinical efficacy of Dapagliflozin, underscoring the necessity for additional data to substantiate its role in managing patients with AHF.

**Clinical trial number:**

Not applicable.

**Supplementary Information:**

The online version contains supplementary material available at 10.1186/s12872-024-04412-x.

## Introduction

Acute Heart Failure (AHF) is characterized by abnormal cardiac function, leading insufficient blood flow to meet the metabolic needs of tissues [1]. This critical condition carries high morbidity and mortality that requires immediate medical intervention to avoid serious complications [1]. Studies indicate a 2-year I mortality rate of 52.8% among AHF patients, underscoring the acute and life-threatening nature of this condition [2]. AHF marks a combination of distressing symptoms such as dyspnea (shortness of breath), orthopnea (difficulty breathing when lying down), lower limb swelling, and clinical manifestations such as increased jugular venous pressure and accumulation of fluid in the lungs [3].

The cornerstone of treatment in the most common presentations of AHF has been diuretic therapy, often combined with vasodilators. According to current international guidelines, intravenous loop diuretics are recommended as the first-line treatment for AHF. Together, these drugs alleviate congestion and improve the stroke volume to promote better oxygen delivery by dilating both arterial and venous vessels [4]. However, approximately one-third of the patients cannot achieve decongestion due to the onset of acute cardiorenal syndrome, CRS-1, which denotes a sudden decline in cardiac function accompanied by reduced kidney function and significant resistance to diuretic therapy [5].

Dapagliflozin, a sodium-glucose cotransporter-2 inhibitor (SGLT2i), is a beacon of hope in the treatment of heart failure. It lowers glucose by blocking the SGLT2 protein located in the proximal convoluted tubule (PCT) of the nephron [6]. This novel antidiabetic agent is a viable therapeutic option for heart failure [7]. Additionally, clinical studies demonstrate a reduction in cardiovascular morbidity and mortality, a general reduction in hospital readmission rates, and improved survival prospects for patients with HF [8] While underlying studies indicate promising potential, the clinical evidence on the utility of SGLT2 inhibitors in this context is still evolving, and there remains an opportunity to expand the current clinical evidence. In this meta-analysis, we aim to assess the safety and efficacy of dapagliflozin in AHF, aiming to clarify its therapeutic role.

## Methods

The meta-analysis followed the guidelines specified in the Preferred Reporting Items for Systematic Reviews and Meta-Analysis (PRISMA) [9].

### Data sources and search strategy

An exhaustive and thorough search was carried out using various electronic databases, including PubMed, Science Direct, and Cochrane Library. Online databases such as ClinicalTrials.gov were carefully reviewed to identify unpublished or gray literature. The search utilized terms such as “Dapagliflozin AND acute heart failure.” Additionally, the references cited in the retrieved papers were thoroughly reviewed manually to discover relevant research. No restrictions on language or publication date were imposed, ensuring a comprehensive and unbiased search. The precise search strategy is shown in (Supplementary Table 1).

### Data synthesis and data extraction

All retrieved studies were imported into the EndNote Reference Library (Version X7.5; Clarivate Analytics, Philadelphia, PA) for duplicate removal and screening. Two independent reviewers (M.H.S. and A.R.) screened the titles and abstracts, followed by a full-text review to ensure adherence to inclusion criteria. Discrepancies were resolved by a third reviewer (S.R).Studies were included if they fulfilled the following criteria: (i) written in English (ii) reported outcomes of interest, (iii) were randomized controlled trials (RCTs). Ongoing trials, letters, case reports, abstracts, reviews, and extension studies were excluded. Relevant information such as the primary author, year of publication, total number of participants, group-specific patient counts, mean age across groups, and baseline comorbidities were systematically collected using an electronic data extraction form. The primary outcome was In-Hospital Cardiovascular mortality in AHF patients. Secondary outcomes were Length of Hospital Stay in Days, 30 Days Hospital Readmission, Change in Weight from Baseline in kg, Hypotension, Hospital Worsening Heart Failure and Renal Failure, Change in GFR at the End of the Study, mL/min/1.73 m^2^.

### Risk of bias and quality assessment

We evaluated the risk of bias in the included RCTs using the Risk of Bias 2 tool (RoB 2) as advised by the Cochrane Collaboration [10]. The studies were rigorously evaluated according to their selection bias, blinding of participants and personnel, blinding of outcome assessment, incomplete outcome data, and selective reporting. Low risk of bias, high risk of bias, and unclear risk of bias (lack of information) are the three categories into which the studies were classified. Two authors, independently assessed the titles and abstracts of the articles and conducted a thorough review of the full texts to determine if the studies met the inclusion criteria. Disparities were settled through discussion with a third author, ensuring the objectivity of the process.

### Statistical analysis

For the statistical analysis, Review Manager (Rev Man Version 5.4.1) from the Cochrane Collaboration in London, UK, was utilized. Dichotomous outcomes were assessed using relative risk (RR) with 95% confidence intervals (95% CIs), while continuous outcomes were analyzed using mean difference (MD). All results were presented with 95% CIs. A random-effects model was applied to combine outcomes, and statistical heterogeneity was evaluated using Higgins’s I² statistic: I² < 50% indicated mild, 50–75% moderate, and > 75% severe heterogeneity [11]. A p-value ≤ 0.05 was considered statistically significant. Additionally, a leave-one-out analysis was conducted for outcomes showing severe heterogeneity.

## Results

### Study selection and characteristics

**Fig. 1:**
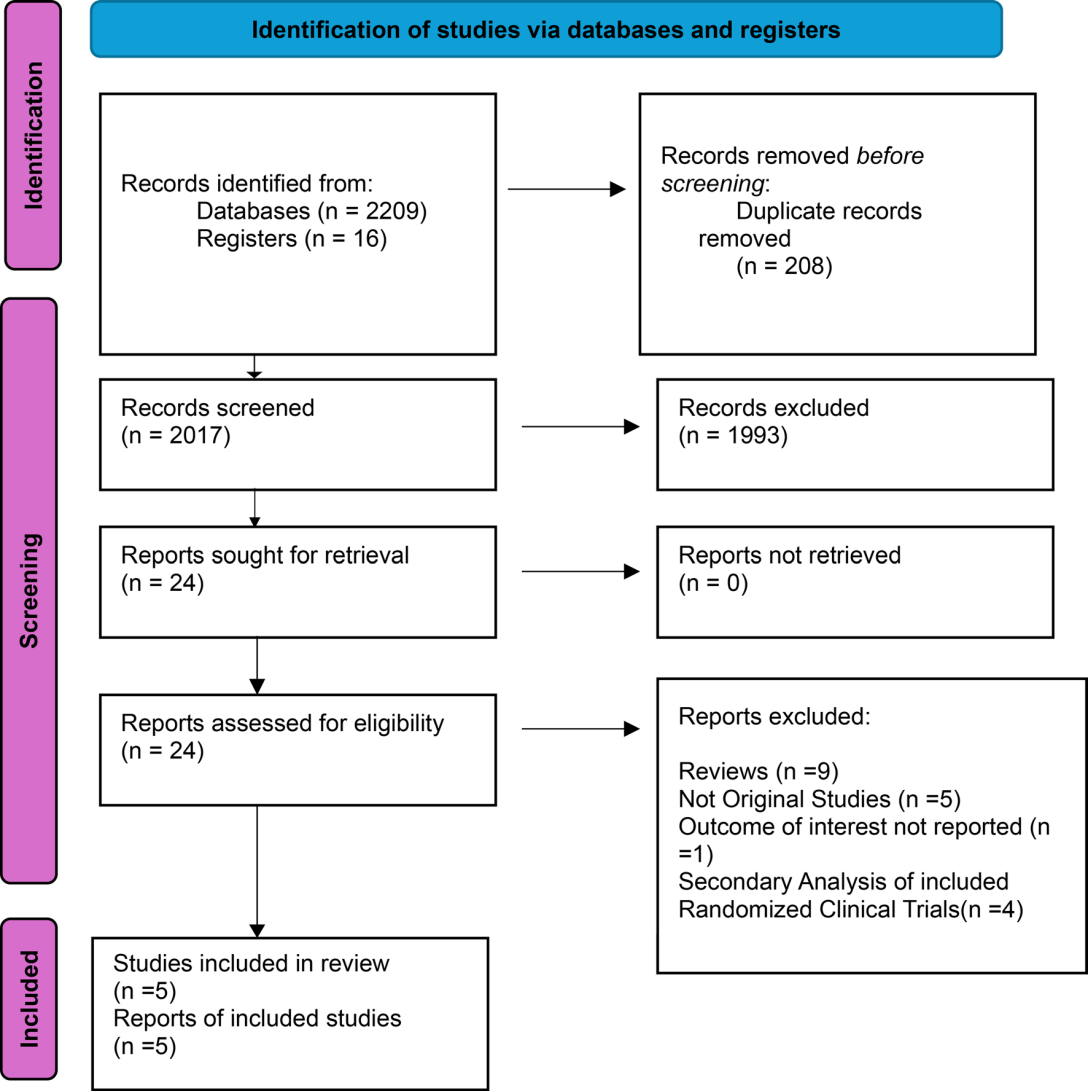
Prisma flow chart

Our comprehensive systematic search of various databases identified 2,225 records. After eliminating duplicates and screening, we identified 5 randomized controlled trials that met our stringent eligibility criteria and were included in this meta-analysis [12–16]. This rigorous selection process ensures the reliability and trustworthiness of our findings. The PRISMA flowchart below (Fig. 1) provides a concise overview of our screening process. The patient population consisted of a total sample size of 912, with 448 in the dapagliflozin group and 464 in the control group, including male and female participants from various age groups within the adult population. The median ages ranged from 68.7 years in the dapagliflozin group to 70.42 years in the control group. The baseline characteristics of the studies included and patients are presented in Tables 1 and 2.

**Table 1:** General characteristics of included studies table

Study name	Study year	Study design	Country	Total sample size	Patients		Primary outcome	Drug dose(mg)	Follow up duration (Days)	Patient category
					Dapagliflozin	Control group				
Emara et al.	2023	Randomized, double-blind study	Egypt	87	45	42	Severity of dyspnea	10 mg	30 Days	Acute heart failure
Zachary et al.	2024	Randomized, open-label study (in multicentre)	USA	238	119	119	Diuretic efficiency	10 mg	Until 5 Days/Hospital Discharge	Acute heart failure
K Charaya et al.	2023	Controlled randomized study	Russia	200	94	106	Acute kidney ijury	10 mg	NA	Acute heart failure
Charaya et al.	2023	Controlled randomized study (in a single center)	Russia	285	140	145	Change in plasma sodium concentration	10 mg	MA	Acute heart failure
Charaya et al.	2022	Controlled randomized study (in a single center)	Russia	102	50	52	Cardiovascular death or hospitalisation for heart failure	10 mg	30 Days	Acute heart failure

**Table 2:** Patient baseline characteristics table

Study name	Patients			Age -yr		Gender M/F		Systolic BP (mmHg)		Diastolic BP (mmHg)		Heart rate (bpm)		Hypertension		DM TYPE 2		Atrial fibrillation		Loop diuretics		ACEIs/ARBs		β Blockers		NT-proBNP (pg/mL)		Glucose (mmol/L)	
	Dapagliflozin	Control group		Dapagliflozin	Control group	Dapagliflozin	Control group	Dapagliflozin	Control group	Dapagliflozin	Control group	Dapagliflozin	Control group	Dapagliflozin	Control group	Dapagliflozin	Control group	Dapagliflozin	Control group	Dapagliflozin	Control group	Dapagliflozin	Control group	Dapagliflozin	Control group	Dapagliflozin	Control group	Dapagliflozin	Control group
Emara et al. 2023	45	42		61.1 (11.8)	63.9 (10)	35/10	27/15	120	115	80	70	82	87	22 (48.9%)	21 (50%)	16 (35.6%)	22 (52.4%)	14 (13.1%)	13 (31%)	31 (68.9%)	31 (73.8%)	21 (46.7%)	16 (38.1%)	-	-	3600	3100	-	-
Zachary et al. 2024	119	119		65 (56–73)	64 (55–74)	78/41	67/52	121	120	-	-	-	-	-	-	84 (71%)	85 (71%)	50 (42%)	49 (41%)			59 (50%)	64 (54%)	68 (58%)	73 (62%)	2277	2927	6.71	6.99
K Charaya et al. 2023	94	106		73 ± 12	75 ± 12	53/42	49/53	131 ± 16.5	130 ± 18	79 ± 8.5	79 ± 9.6	94 ± 19	98 ± 22	90 (96%)	101 (99%)	27 (29%)	35 (34%)	61 (65%)	72 (71%)	59 (63%)	55 (54%)	71 (76%)	72 (71%)	61 (65%)	65 (64%)	-	-	7.4 ± 3	7.5 ± 3
Charaya et al. 2023	140	145		72 ± 12	75 ± 13	78/62	73/72	130 ± 16	128 ± 17	78.5 ± 8	78.9 ± 9	94.2 ± 20	96 ± 21	120 (86%)	126 (87%)	44 (31%)	56 (38%)	92 (66%)	96 (66%)	-	-	-	-	-	-	5100	4191	6.7	6.6
Charaya et al. 2022	50	52		72.6 ± 12.2	74.2 ± 11.3	29/21	27/25	132.9 ± 17.9	130.1 ± 21.1	78.72 ± 8.7	78.5 ± 11	91.8 ± 17.85	93.6 ± 18.92	46 (92%)	48 (92%)	15 (30%)	16 (30%)	25 (50%)	30 (57%)	33 (66%)	32 (62%)	37 (74%)	35 (67%)	28 (56%)	33 (63%)	5333	4381	7	6.45

### Risk of bias assessment

**Fig. 2:**
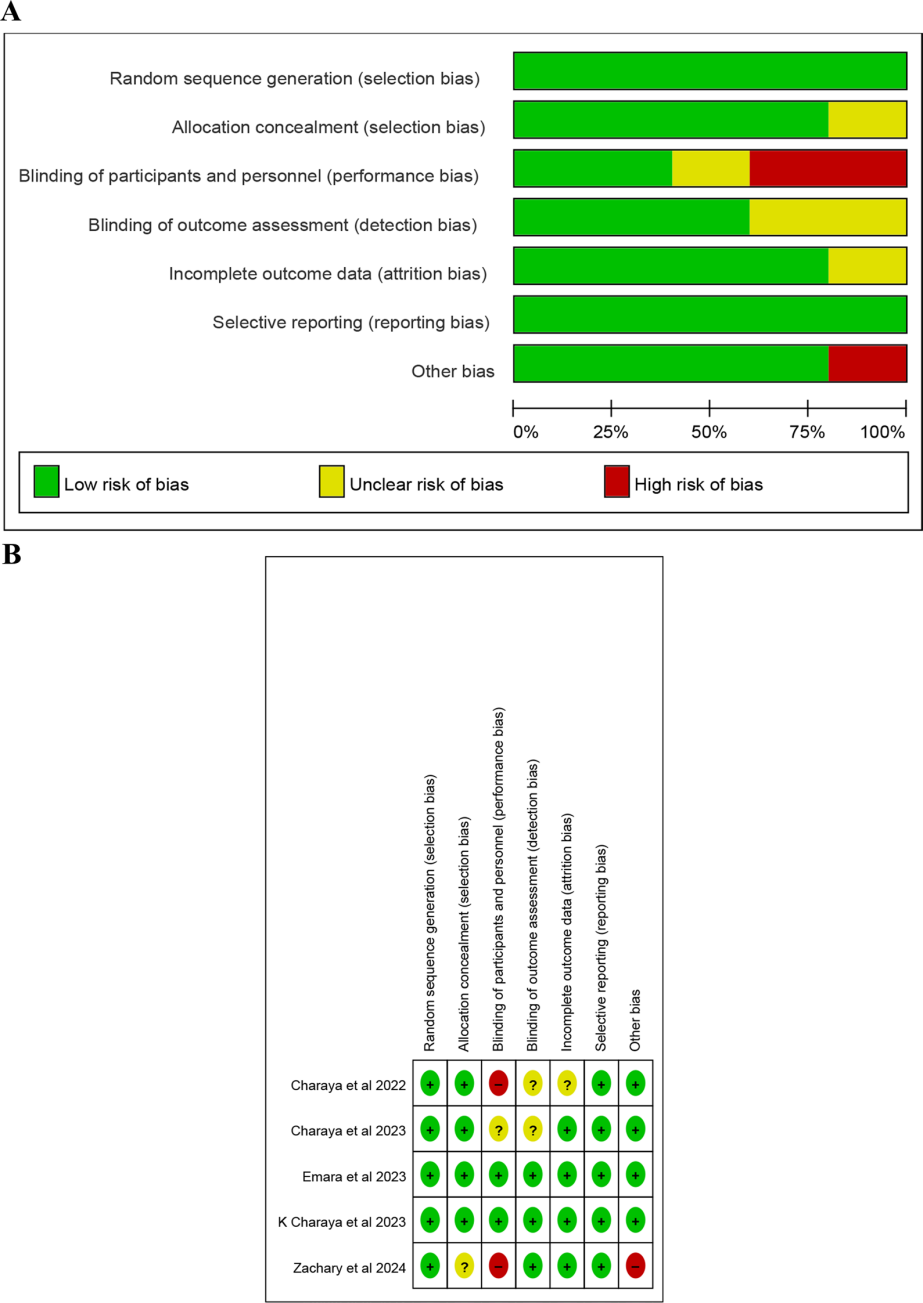
**A** risk of bias graph. **B**. risk of bias summary

We meticulously assessed the risk of bias using the Risk of Bias 2 tool (RoB 2). Most studies showed a low risk of bias for random sequence generation and allocation concealment (Fig. 2A and B). The detailed risk of bias assessment is depicted in Supplementary Table 2. This thorough assessment ensures the integrity and robustness of our study.

### Primary outcome

#### In-hospital cardiovascular mortality

**Fig. 3:**
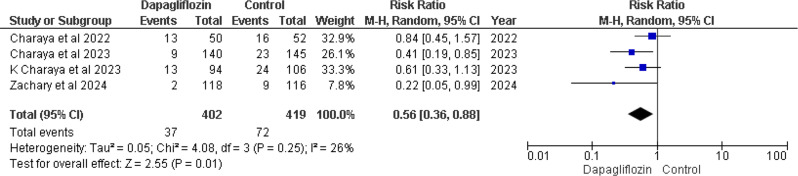
Forest plot of In-hospital cardiovascular mortality

Four out of five studies [12, 14–16] reported In-Hospital Cardiovascular mortality. Pooled analysis revealed that treatment regimens involving dapagliflozin showed a notably reduced risk of death compared to the control group. The combined result of four studies was RR 0.56 (95% CI 0.36–0.88), with a p-value of 0.01 and I² = 26% (Fig. 3).

### Secondary outcomes

#### Length of hospital stay in days

**Fig. 4:**
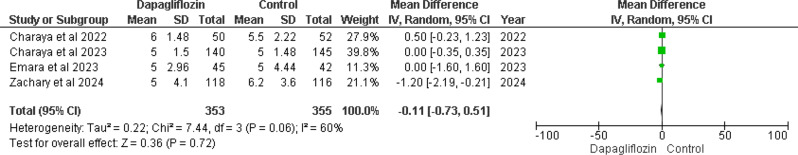
Forest plot of length of hospital stay in days

Four of the included studies [12, 13, 15, 16] assessed hospital stay duration in days. The MD was − 0.11 (95% CI -0.73 to 0.51), yielding a non-significant p-value of 0.72 and demonstrating moderate heterogeneity (I² = 60%). This suggests a slight reduction in hospital stay length in the dapagliflozin group compared to controls. However, the wide 95% confidence interval indicates uncertainty in the precise effect estimate. The observed heterogeneity, while not statistically significant (*p* = 0.06), suggests heterogeneity across studies (Fig. 4).

### 30-day hospital readmission

**Fig. 5:**
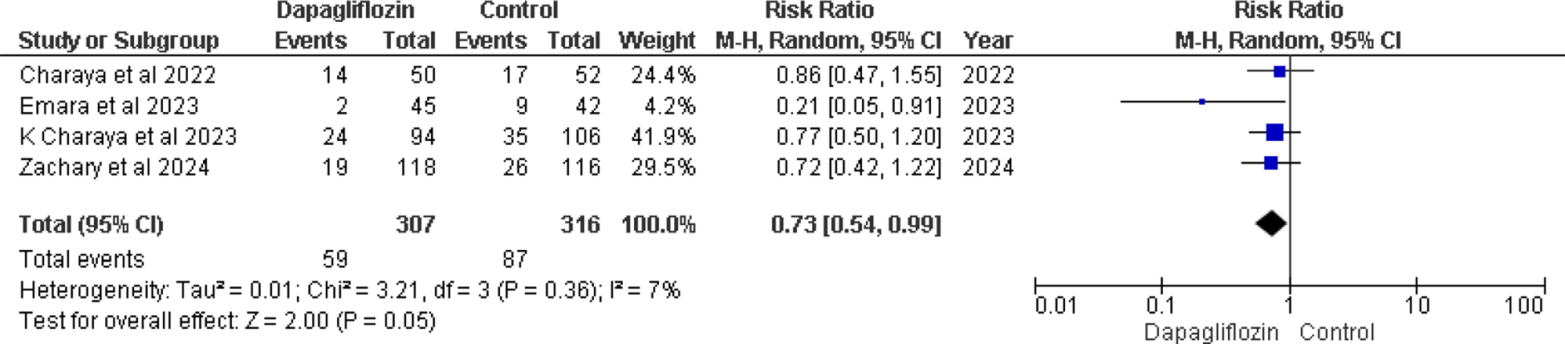
Forest plot of 30 days hospital readmission

Four studies [12–14, 16] provided data on 30-day hospital readmission rates, indicating a notable decrease in readmission risk associated with dapagliflozin. The pooled analysis revealed a RR of 0.73 (95% CI 0.54–0.99), with a marginally significant p-value of 0.05 and low heterogeneity (I² = 7%) (Fig. 5).

### Change in weight from baseline in kg

**Fig. 6:**
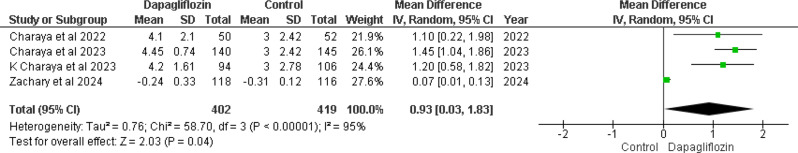
Forest plot of change in weight from baseline in kg

The pooled analysis of four out of five studies [12, 14–16] revealed a (MD) of 0.93 (95% CI 0.03–1.83), with a significant p-value of 0.04 and substantial heterogeneity (I² = 95%) in weight change from baseline between the dapagliflozin and control groups (Fig. 6). This significant heterogeneity (*p* < 0.00001, I² = 95%) prompts the need for sensitivity analysis, including leave-one-out analysis, to further explore these differences.

### Leave-one-out analysis

High heterogeneity prompted a sensitivity analysis where removing Zachary et al. 2024 (11) reduced it from I² = 95% to I² = 0%. This showed a more consistent effect across studies, with an improved MD of 1.34 (95% CI 1.02–1.66) and a p-value < 0.0001 (Supplementary Fig. 1).

### Hypotension

**Fig. 7:**
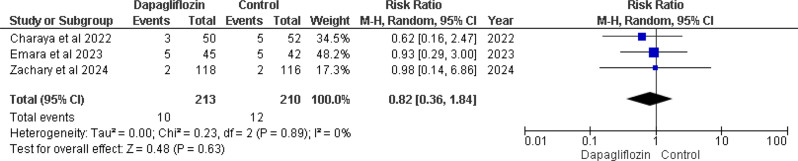
Forest plot of hypotension

Three of the five studies included in the analysis reported this outcome [12, 13, 16]. The pooled analysis showed an overall (RR) of 0.82 (95% CI 0.36–1.84), with a non-significant p-value of 0.63 and no heterogeneity (I² = 0%). This suggests a trend towards a lower risk of hypotension in the dapagliflozin group compared to the control group. Despite the lack of significant heterogeneity, the p-value of 0.63 suggests that there are no statistically significant differences in the occurrence of hypotension across the groups. (Fig. 7).

### In-hospital worsening

**Fig. 8:**
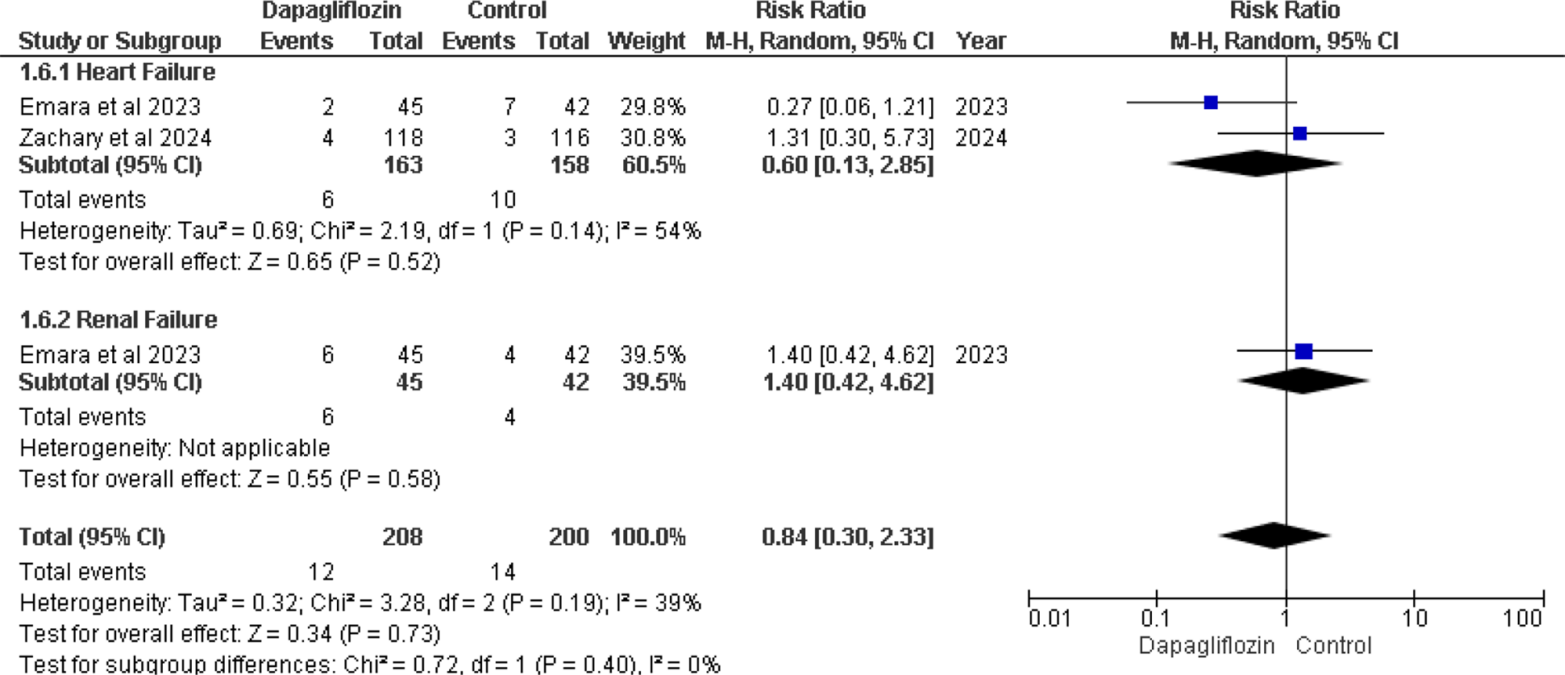
Forest plot of In hospital worsening heart failure and renal failure

Emara et al. 2023 and Zachary et al. 2024 [12, 13] cases of in-hospital worsening heart failure. The overall effect for heart failure showed RR 0.60 (95% CI 0.13–2.85), with a p-value of 0.52 and I² = 54%. Emara et al. 2023 also reported in-hospital worsening renal failure. The overall effect for renal failure showed RR 1.40 (95% CI 0.42–4.62), with a p-value of 0.58. Both p-values showed no statistically significant differences (Fig. 8).

### Change in GFR at the end of the study (mL/min/1.73 m²)

**Fig. 9:**
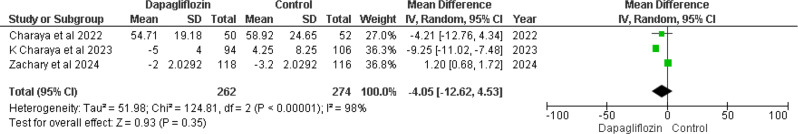
Forest plot of change in GFR at end of study, mL/min/1.73 m2

Three out of five included RCTs [12, 14, 16] reported changes in GFR at the end of the study. The pooled result analysis showed MD -4.05 (95% CI -12.62 to 4.53), with a p-value of 0.35 and I² = 98% (Fig. 9). The p-value suggests that the results were non-significant between the dapagliflozin and control groups. Significant heterogeneity was present (I² = 98%, p-value < 0.00001), necessitating a sensitivity analysis by performing a leave-one-out analysis.

### Leave-one-out analysis

After excluding Zachary et al. 2024 [12], results improved, with heterogeneity decreasing from I² = 98% to I² = 22%. The results showed MD -8.54 (95% CI -11.98 to -5.10), with a p-value < 0.00001 (Supplementary Fig. 2). This suggests significant trends toward changes in GFR at the end of the study for the dapagliflozin group compared to the control group.

## Discussion

Our meta-analysis explored the therapeutic potential of dapagliflozin in AHF by examining data from five randomized controlled trials, encompassing 912 patients. We assessed various primary and secondary outcomes to thoroughly evaluate the drug’s efficacy and safety. In-Hospital Cardiovascular mortality was our primary endpoint, while secondary endpoints included hospital stay duration, 30-day readmission rates, changes in weight, incidence of hypotension, and GFR alterations. Our findings reveal dapagliflozin shows promise in reducing In-Hospital Cardiovascular mortality, hospital readmissions, and weight in AHF though some secondary outcomes such as hospital stay duration, hypotension risk did not consistently favor dapagliflozin. Overall, dapagliflozin demonstrated beneficial effects compared to alternative treatments. However, caution is warranted due to study limitations such as small sample sizes and potential biases in the included trials. Despite these limitations, our analysis underscores dapagliflozin’s potential in reducing In-Hospital Cardiovascular mortality, heart failure exacerbations, hypotension risks, and hospitalizations, alongside effects on weight and GFR. Notably, our study did not find significant benefits related to renal function based on the available data.

AHF management involves several treatment options, each with distinct benefits and drawbacks. Diuretics are a cornerstone in AHF management, crucial for reducing congestion and alleviating fluid overload symptoms by promoting vasodilation and increasing sodium and water excretion. However, diuretics require close monitoring to mitigate risks associated with electrolyte imbalances, which can lead to hospitalization and increased mortality rates [17, 18]. Vasodilators, including ACE inhibitors (ACE-Is) and nitrates, improve hemodynamics and enhance exercise capacity. Yet, ACE-Is can cause profound hypotension and renal complications, while nitrates are associated with tolerance and adverse effects such as headaches and hypotension [19, 20]. Calcium channel blockers [CCBs), despite their anti-arrhythmic benefits and potential to enhance diastolic capacity, are limited by their cardio-depressive effects and restricted usage in heart failure patients [21, 22]. In the quest for new therapies to manage AHF effectively, dapagliflozin has emerged as a promising option. As a sodium-glucose co-transporter type 2 (SGLT2) inhibitor, dapagliflozin primarily acts on renal receptors to increase glucose excretion, a mechanism widely utilized in the management of type 2 diabetes [23]. In the context of AHF, dapagliflozin enhances diuresis, optimizes ventricular loading conditions, and reduces preload. Additionally, it influences adipose tissue distribution, decreases arterial stiffness and blood pressure, and promotes cardiac and lipid metabolism through various adipokines [24–27].

Various studies have explored the impact of dapagliflozin on mortality rates in patients with heart failure. Research consistently indicates that dapagliflozin is linked to a reduced risk of mortality, primarily from cardiovascular causes such as heart failure and sudden cardiac death [28]. Although our study findings are limited to cardiac deaths, dapagliflozin’s effect on non-cardiac deaths is also noteworthy. Other studies have revealed that dapagliflozin reduces non-cardiac deaths, including those from malignancy and infections [29]. This mortality benefit extends beyond patients with HF to those with co-existing conditions such as diabetes or chronic kidney disease [30]. Consequently, dapagliflozin appears to be effective in reducing both cardiac and non-cardiac deaths [31]. These results align with our study, which examined the effect of dapagliflozin on In-Hospital Cardiovascular mortality. Additionally, research has shown that dapagliflozin use is associated with shorter hospital stays, particularly when initiated early in the course of treatment. It has also been observed that dapagliflozin reduces the length of hospital stays in individuals with severe heart failure, leading to a decreased need for readmission [32, 33]. Our findings were consistent with previous research in this regard [34]. However, the impact of dapagliflozin on hospital stay duration in our study did not show a significant reduction (*p* = 0.72]. In terms of readmissions, dapagliflozin was found to reduce the risk of 30-day readmissions compared to placebo. This benefit was more pronounced in patients with severe heart failure and diabetes, although non-diabetic patients and those with milder disease also experienced advantages from this medication [34].

Dapagliflozin exerts a significant impact on both heart failure (HF) and renal function, influencing multiple physiological pathways. By enhancing ventricular function, dapagliflozin reduces the risk of HF worsening across various patient groups, regardless of diabetes status [35, 36]. Studies have consistently shown lower rates of HF exacerbation and hospitalization among dapagliflozin users, highlighting its role in improving clinical outcomes in HF patients [37]. However, conflicting results have been observed regarding its impact on renal function [36]. Some trials indicate that dapagliflozin reduces the risk of worsening renal function in patients with a GFR below 25 ml/min/1.73 m², which contrasts with our findings [38]. Other studies support the notion that dapagliflozin can slow renal decline, emphasizing its potential for renal protection [37]. Our study also demonstrated dapagliflozin’s ability to minimize the incidence of hypotension, corroborated by research on lipoprotein-associated hypotension [39]. Despite concerns about orthostatic hypotension in specific contexts, the overall evidence suggests that dapagliflozin does not significantly increase the risk of hypotension [40–42]. Heterogeneity in our findings, particularly regarding weight change and GFR endpoints influenced by studies like Zachary et al. [12], underscores the need for adjustments to enhance consistency across diverse populations.

Our meta-analysis did not reveal a significant reduction in hospital stay duration for patients treated with dapagliflozin compared to controls. This outcome aligns with dapagliflozin’s known effects on diuresis and volume reduction, which can lead to quicker stabilization of patients with AHF [36]. The early initiation of dapagliflozin could facilitate faster clinical improvement, thereby reducing hospital stay lengths. Additionally, dapagliflozin significantly reduced the risk of 30-day readmissions, particularly in patients with severe heart failure and diabetes. The reduction in readmission rates is likely due to the sustained hemodynamic and metabolic benefits of dapagliflozin, which include improved ventricular function and reduced fluid overload [37]. These effects help prevent acute decompensation post-discharge, a common cause of early readmissions. Moreover, our analysis showed significant weight reduction in patients receiving dapagliflozin. Weight reduction can be attributed to dapagliflozin’s diuretic effects, leading to decreased fluid retention, and its impact on glucose and lipid metabolism, contributing to fat loss [25]. This is particularly beneficial in heart failure management as it reduces the workload on the heart.

Dapagliflozin also demonstrated a significant reduction in the incidence of hypotension among its users. Its role in reducing preload and afterload without causing excessive vasodilation likely contributes to its lower risk of hypotension compared to other heart failure medications [24, 26]. This finding is crucial, as hypotension can limit the use of certain therapies in heart failure patients. However, the effects on GFR were mixed, with no statistically significant changes overall. Dapagliflozin has a complex impact on renal function. Initially, it can cause a mild reduction in GFR due to diuresis and reduced intraglomerular pressure. However, long-term benefits include renal protection through mechanisms such as reduced hyperfiltration and improved glycemic control. The lack of significant GFR change in our meta-analysis could be due to the short follow-up periods in the included studies, which were insufficient to capture long-term renal benefits.

Our study acknowledges several limitations that impact our findings. The small sample size may compromise the robustness and generalizability of our results, while the lack of adequate studies precluded meta-regression analysis, limiting our ability to adjust for confounding variables. Additionally, limited data availability may have restricted our comparisons of various medications, hindering our assessment of their comprehensive effects. We were also unable to evaluate adverse events or cost-effectiveness, both vital in shaping dapagliflozin’s clinical profile. Furthermore, the inconsistent reporting of renal safety endpoints, such as worsening renal function (WRF), oligoanuria, and the initiation of renal replacement therapy, restricted our analysis of dapagliflozin’s renal impact. Future research should focus on conducting larger studies that consistently report renal safety endpoints and evaluate adverse events and cost-effectiveness. Additionally, it should explore the effects of different dosages of dapagliflozin to provide a comprehensive understanding of its role in AHF management.

## Conclusion

Our study underscores the significance of dapagliflozin in the management of AHF. We demonstrated the superior efficacy of dapagliflozin compared to the control group in treating AHF, highlighting its pivotal role in AHF therapy. However, further research is warranted to deepen our understanding of this drug in the context of AHF. Future studies should consider larger sample sizes, explore different dosage regimens, and conduct multiple comparisons to comprehensively evaluate dapagliflozin’s therapeutic potential. Additionally, it is essential for upcoming research to include discussions on cost-effectiveness and conduct detailed safety analyses. Furthermore, evaluating the efficacy of various doses of dapagliflozin should be a priority for future investigations. Such research efforts will help solidify the place of dapagliflozin in the therapeutic arsenal against AHF, potentially improving outcomes for a broad spectrum of patients.

## Electronic supplementary material

Below is the link to the electronic supplementary material.

Supplementary Material 1

## Data Availability

The authors affirm that the data underpinning the findings of this study is accessible online and including within the supplementary material.
