# Supplementary material for: Dapagliflozin in acute heart failure management: a systematic review and meta-analysis of safety and effectiveness
Source: BMC Cardiovasc Disord. 2024 Dec 28;24:749. doi: 10.1186/s12872-024-04412-x (PMC11681638; doi:10.1186/s12872-024-04412-x)

**Supplementary Material**

**Supplementary Table 1.** Search Strategies for different Databases

| **Database** | **Search String** |
| --- | --- |
| PubMed/MEDLINE  (119 Results) | ("dapagliflozin"[Supplementary Concept] OR "dapagliflozin"[All Fields] OR "dapagliflozin s"[All Fields]) AND (("acute"[All Fields] OR "acutely"[All Fields] OR "acutes"[All Fields]) AND ("heart"[MeSH Terms] OR "heart"[All Fields] OR "hearts"[All Fields] OR "heart s"[All Fields]) AND "faiure"[All Fields]) |
| Cochrane Library  (119 Results) | Dapagliflozin AND acute heart failure |
| Science Direct  (1971 Results) | Dapagliflozin AND acute heart failure |
| Clinical Trial Govt  (16 results) | Intervention: Dapagliflozin;Condition: Acute Heart Failure |

**Supplementary Table 2. Risk of Bias Table.**

|  | Cochrane Risk-of-Bias Tool | | |  |
| --- | --- | --- | --- | --- |
|  | Bias | Risk of bias | Author judgement | |
| Charaya et al 2023 | Random sequence generation (selection bias) | Low Risk | Patients were randomized to a main group (standard therapy supplemented with dapagliflozin) or a control group (standard therapy for ADHF). | |
|  | Allocation concealment (selection bias) | Low Risk | . Patients were randomized using a sealed envelope method. Therefore, it's likely that allocation concealment was effectively implemented, minimizing biased allocation to interventions. | |
|  | Blinding of participants and personnel (performance bias) | unclear | The study did not specify whether blinding of participants and personnel was implemented. There was no explicit mention of blinding in the study design or methodology | |
|  | Blinding of outcome assessment (detection bias) | unclear | The study did not provide explicit information on whether the outcome assessors were blinded to the intervention allocation. Without this information, it is unclear whether there was blinding of outcome assessors to reduce the risk of detection bias. | |
|  | Incomplete outcome data (attrition bias) | Low Risk | Incomplete outcome data were handled appropriately, minimizing the risk of attrition bias. | |
|  | Selective reporting (reporting bias) | Low Risk | There is no evidence of selective outcome reporting, reducing the risk of reporting bias. | |
|  | Other bias | Low Risk | No other biases were identified in the study that could significantly impact the results. | |
| K Charaya et al 2023 | Random sequence generation (selection bias) | Low Risk | Subjects were randomly assigned to the Dapagliflozin or placebo group. | |
|  | Allocation concealment (selection bias) | Low Risk | Although the allocation concealment process was not explicitly described, the trial was double-blind, and patients and personnel were unaware of individual patient identification and treatment assignments. Therefore, it's likely that allocation concealment was effectively implemented, minimizing biased allocation to interventions. | |
|  | Blinding of participants and personnel (performance bias) | Low Risk | The study was described as "double-blind," indicating that both participants and personnel were blinded to the treatment allocation. | |
|  | Blinding of outcome assessment (detection bias) | Low Risk | tTe study was "double-blind," which typically includes blinding of outcome assessors, decreasing the risk of detection bias. | |
|  | Incomplete outcome data (attrition bias) | Low Risk | Incomplete outcome data were handled appropriately, minimizing the risk of attrition bias. | |
|  | Selective reporting (reporting bias) | Low Risk | There is no evidence of selective outcome reporting, reducing the risk of reporting bias. | |
|  | Other bias | Low Risk | No other biases were identified in the study that could significantly impact the results. | |
| Emara et al 2023 | Random sequence generation (selection bias) | Low Risk | Eligible patients were randomized in a 1:1 ratio to receive either dapagliflozin or placebo. . | |
|  | Allocation concealment (selection bias) | Low Risk | The randomization schedule was concealed in sequentially numbered envelopes, indicating allocation concealment was implemented. Therefore, allocation concealment was effectively implemented, minimizing biased allocation to interventions. | |
|  | Blinding of participants and personnel (performance bias) | Low Risk | The study mentions that treatment assignment was masked from all patients and care providers indicating blinding of participants and personnel. | |
|  | Blinding of outcome assessment (detection bias) | Low Risk | The outcome assessment was undertaken by a qualified researcher who was not involved in patient care indicating blinding of outcome assessment. | |
|  | Incomplete outcome data (attrition bias) | Low Risk | The primary outcome was assessed for all randomized patients who were included in the final analysis, suggesting appropriate handling of incomplete outcome data,, minimizing the risk of attrition bias. | |
|  | Selective reporting (reporting bias) | Low Risk | There is no evidence of selective outcome reporting, reducing the risk of reporting bias. | |
|  | Other bias | Low Risk | No other biases were identified in the study that could significantly impact the results. | |
| Charaya et al 2022 | Random sequence generation (selection bias) | Low Risk | The study mentioned the use of the 'envelope' method for randomization, where patients were assigned a group using sequentially-numbered sealed opaque envelopes containing allocation cards. This method helps in reducing selection bias by ensuring random allocation of participants to treatment groups. | |
|  | Allocation concealment (selection bias) | Low Risk | Although the allocation concealment process was not explicitly described, the use of sealed opaque envelopes for randomization suggests an attempt at allocation concealment. Proper allocation concealment helps in preventing selection bias by ensuring that the treatment allocation is not influenced by the researchers' knowledge or preferences. | |
|  | Blinding of participants and personnel (performance bias) | High Risk | The study mentioned that it was an open-label trial, indicating that participants and personnel were not blinded to the treatment allocation. Lack of blinding can introduce performance bias as participants and personnel may behave differently based on their knowledge of the treatment received. | |
|  | Blinding of outcome assessment (detection bias) | Unclear | The study did not mention blinding of outcome assessment, which could introduce detection bias. | |
|  | Incomplete outcome data (attrition bias) | Unclear | The study did not provide specific details on how incomplete outcome data were handled. | |
|  | Selective reporting (reporting bias) | Low Risk | There is no evidence of selective outcome reporting, reducing the risk of reporting bias. | |
|  | Other bias | Low Risk | No other biases were identified in the study that could significantly impact the results. | |
| Zachary et al 2024 | Random sequence generation (selection bias) | Low Risk | The study mentions that patients were randomized, but specific details on the sequence generation method are not provided​. | |
|  | Allocation concealment (selection bias) | Unclear | The study does not provide specific details on the method used for allocation concealment​. | |
|  | Blinding of participants and personnel (performance bias) | High Risk | The study was open-label, meaning that both participants and personnel were aware of the treatment allocation​. | |
|  | Blinding of outcome assessment (detection bias) | Low Risk | Secondary and safety outcomes were adjudicated by a blinded committee, which helps mitigate detection bias​ | |
|  | Incomplete outcome data (attrition bias) | Low Risk | The study handled incomplete outcome data appropriately by conducting a proportional odds model adjusted for baseline weight, and by ensuring that outcomes were adjudicated by a blinded committee​ | |
|  | Selective reporting (reporting bias) | Low Risk | There is no evidence of selective outcome reporting, reducing the risk of reporting bias. | |
|  | Other bias | High Risk | Yes, the study acknowledges that the open-label design could introduce unmeasured biases and mentions limitations due to the moderate sample size and specific conditions during the COVID pandemic​ | |
|  | Other bias | Low Risk | No other biases were identified in the study that could significantly impact the results. | |

**Supplementary Figure 1. Leave One Out Analysis for Change in Weight from Baseline in KG**


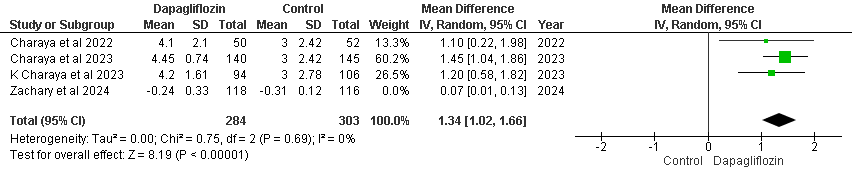


**Supplementary Figure 2. Leave One Out Analysis for Change in GFR at End of Study, mL/min/1.73 m2**


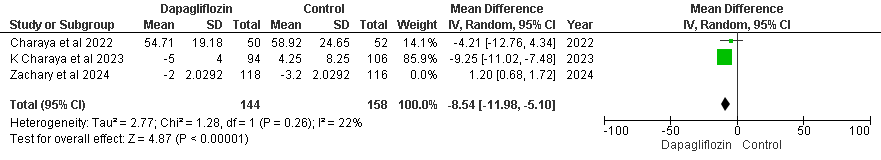

Supplement: Supplementary file 1 — Supplementary Material 1 [file 12872_2024_4412_MOESM1_ESM.docx]
